# Development and psychometric evaluation of an instrument to assess Knowledge, Attitude and Practice of Family Caregivers at Preventing Pressure Injuries (KAP-PI) in Indonesian community-dwelling older adults

**DOI:** 10.1186/s12912-022-00957-4

**Published:** 2022-08-11

**Authors:** Sheizi Prista Sari, Irma H. J. Everink, Christa Lohrmann, Yufitriana Amir, Eka Afrima Sari, Ruud J. G. Halfens, Dimitri Beeckman, Jos M. G. A. Schols

**Affiliations:** 1 Faculty of Nursing, Universitas Padjadjaran, Jl. Raya Sumedang KM. 21 Jatinangor, Bandung, West Java Indonesia; 2 Department of Health Services Research, Care and Public Health Research Institute (CAPHRI), Maastricht University, Duboisdomein 30, 6229 GT Maastricht, The Netherlands; 3 Department of Nursing Science, Medical University of Graz, Graz, Austria; 4 Faculty of Nursing, Universitas Riau, Pekanbaru, Indonesia; 5 Skin Integrity Research Group (SKINT), Department of Public Health and Primary Care, University Centre for Nursing and Midwifery, Ghent University, Ghent, Belgium; 6 School of Health Sciences, Örebro University, Örebro, Sweden; 7 School of Nursing and Midwifery, Royal College of Surgeons in Ireland (RCSI), University of Medicine and Health Sciences, Dublin, Ireland; 8 Research Unit of Plastic Surgery, Department of Clinical Research, Faculty of Health Sciences, University of Southern Denmark, Odense, Denmark; 9 School of Nursing and Midwifery, Monash University, Clayton, Australia; 10 Department of Family Medicine, Care and Public Health Research Institute (CAPHRI), Maastricht University, Maastricht, The Netherlands

**Keywords:** Knowledge, Attitude, Practice, Pressure injury, Pressure ulcer, Prevention, Family caregivers, Psychometric evaluation, Community nurses

## Abstract

**Background:**

The prevalence of pressure injuries among community-dwelling older adults in countries worldwide is still a serious problem. In Indonesia, older adults mostly rely on family members for (medical) care. Therefore, involving family members in the prevention and treatment of pressure injuries (PIs) could potentially decrease its prevalence rates. However, family members are usually not trained for such tasks. Hence, it is essential to first get more insight into the current state of affairs on family members’ knowledge, attitude and actual practice of preventing PIs. Due to the lack of an existing instrument to measure knowledge, attitude and practice of family caregivers in preventing PIs, this study focuses on the development and evaluation of psychometric properties of such an instrument.

**Methods:**

Three phases of instrument development and evaluation were used, including item generation, instrument construction and psychometric testing of the instrument. A total of 372 family caregivers of community-dwelling older adults who randomly selected participated in this study. Principal factor analysis, confirmatory factor analysis and Cronbach’s alpha were performed to evaluate factor structure and internal consistency of the Knowledge, Attitude and Practice of Family Caregivers at Preventing Pressure Injuries (KAP-PI) instrument.

**Results:**

The final version of the KAP-PI-instrument consists of a 12-item knowledge domain, a 9-item attitude domain, and a 12-item practice domain with Cronbach’s Alpha values of 0.83, 0.93 and 0.89, respectively. The instrument appeared to be both reliable and valid.

**Conclusion:**

The KAP-PI instrument can be used in family nursing or community nursing practice, education, and research to assess knowledge, attitude and practice of pressure injury prevention of family caregivers.

**Supplementary Information:**

The online version contains supplementary material available at 10.1186/s12912-022-00957-4.

## Introduction

Research has shown that the prevalence of pressure injuries (PIs) among community-dwelling older adults in countries around the world is still a serious problem [1–3]. The European Pressure Ulcer Advisory Panel, National Pressure Injury Advisory Panel and Pan Pacific Pressure Injury Alliance (2019) defined that “A pressure injury is a localized damage to the skin and or underlying tissue, which usually occurs over a bony prominence due to pressure or pressure combined with shear” [1]. A study in the United States found that patients who had a PI on admission to the hospital were dominated (70.6%) by older adults (mean age 72.7 years) living at home [2]. Another study in the United States noted that PI were associated with greater number of older adult living at home re-admissions to the hospital [3]. Similarly, a study in United Kingdom reported numbers of community-dwelling older adults with pressure injuries per 1000 were 1.64 for those aged 65 – 74 and 5.75 for aged ≥ 74 [4].

Indonesia is a country that is also facing challenges related to PIs, due to its ageing population [5]. Indonesia has a population of 273.5 million people [6, 7], of which almost 10% are older adults (60 +) and the majority of these older adults live at home with their families [8]. Nursing homes or other long-term care institutions do exist, but are in minimal number [8, 9]. Also, leaving parents in nursing homes is still taboo for Indonesian people [10]. Older adults who live at home can also receive community care, but it appears that this formal care option is also not being used very often [5, 8]. Therefore, family members are often the ones taking care of older adults with care dependency. They not only take care of activities of daily living their relatives need, such as washing and dressing, but also of more complex (medically-oriented) tasks. However, the result of a national survey from Statistics Indonesia (Badan Pusat Statistik) shows that about 40% of older adults and family caregivers in Indonesia treat their older relatives with non-prescribed or traditional medicine, or do not treat diseases at all [8]. Even though the majority of older adults (78.8%) had health insurance, less than half of them (45.7%) actually seeks formal care [5]. Sometimes, community health volunteers help older adults and their family caregivers and/ or motivate them to seek formal care.

However, community nurses in Indonesia are usually primarily responsible for public health in their work area. They are expected to actively come to the community to increase the accessibility of older adults living in the community to proper care and further involve family members in preventing and treating PIs [5, 11, 12]. Involving family members in the prevention and treatment of PIs could potentially decrease its prevalence rates [11–14], especially among older adults living at home with a high risk of developing PIs. These are mainly older adults with limited mobility [5, 15, 16], a stroke history [5, 17], and nutritional problems [18]. However, little is known about what family members actually know about PI.

Preventing and treating PIs is a complex task, and family members are usually not trained for such tasks [5, 15, 19]. Besides the fact that not much is known about the knowledge family members have about PIs, less is also known about the attitude of family caregivers towards PI prevention and treatment. Knowledge and attitude appear to be positively associated with the actual practice of preventing illnesses [20–23].

To decrease prevalence of PIs in Indonesian community-dwelling older adults, it seems that a strategy focused on their family caregivers could be beneficial. However, to develop a targeted strategy, it is essential to first get more insight into the current state of affairs on family members’ knowledge, attitude and actual practice of preventing PIs. To assess this, a valid and reliable instrument is needed. After a literature search focused on finding a standardized or published instrument that measures family caregivers' knowledge, attitude, and practice on PI prevention, it appeared that hardly any information was available. Only one study was found that reported on the psychometric properties of an instrument measuring knowledge on PI prevention [24].

Due to the lack of an existing instrument to measure knowledge, attitude and practice of family caregivers in preventing PIs, the objective of this study was to develop such an instrument and to describe its psychometric properties. In this study, the steps that were taken to develop and test the instrument are described, as well as the content of the final version of instrument for use in a population of community-dwelling older adults in Indonesia.

## Objective

This study describes the development and psychometric evaluation of an instrument to measure knowledge, attitude and practice of family caregivers at preventing PIs (KAP-PI) among community-dwelling older adults in Indonesia.

## Method

**Fig. 1:**
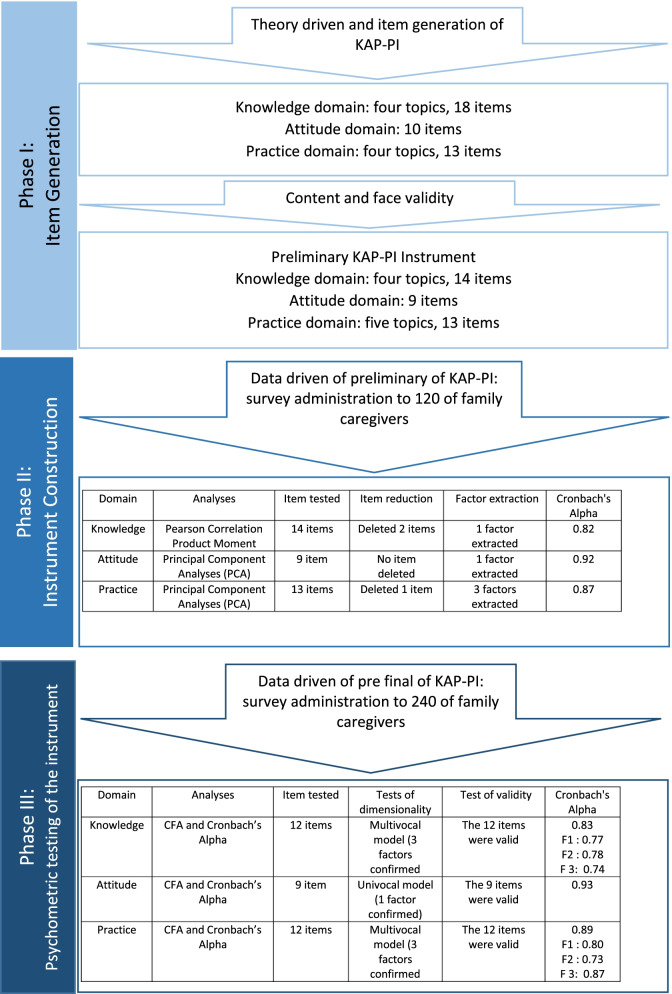
The process of developing and psychometric evaluation of KAPI-PI instrument

Three phases of instrument development and evaluation described by Boateng et al. (2018) were used, including 1) item generation; 2) instrument construction, and 3) psychometric testing of the instrument [25]. Figure 1 describes the entire process of developing and psychometric evaluation of KAPI-PI instrument.

### Phase I: Item generation

#### Item generation

First, we specified the three domains we wanted to measure: knowledge (K), attitude (A), and practice (P) of family caregivers in preventing PIs among community-dwelling older adults in Indonesia. To develop items covering the three domains, authors SPS and EAS conducted a literature search. They independently identified relevant topics for measuring family caregivers’ knowledge, attitudes, and practices for preventing PIs in community-dwelling older adults. The literature review was conducted using the following questions: 1) What is the necessary information about PI prevention that family caregivers should know; 2) What attitude toward PI prevention should family caregivers have; and 3) What should family caregivers actually do to prevent their older relative from getting a PI? After a detailed review of the international PI guideline developed by the National Pressure Ulcer Advisory Panel (NPUAP), The European Pressure Ulcer Advisory Panel (UPUAP) and Pan Pacific Pressure Injury Alliance (PPPIA) [26], the two authors compared and merged their findings. Considering that the guideline is written primarily for health professionals in clinical practice, a review of family nursing books [27–29] was also conducted to narrow the findings in the family care function, resulting in the themes per domain shown in Fig. 1.

After determining the topics in the three domains, the authors proceeded to generating questions per topic. The design of the questions followed the questionnaire design guide explained by Bourke et al. [30]. For the items in the knowledge domain, multiple-choice questions with only one correct answer were developed [31]. For the items in the attitude domain, statements were developed with response options on a Likert scale ranging from 1 (strongly disagree) to 4 (strongly agree) [32]. Statements were also developed in the practice domain. An example is “I help the older relative to move when he is bedridden”. The statements in the practice domain also include a response option on a Likert scale including the answer options ‘never’ (the activity has never been done), ‘sometimes’ (the activity is done 1 to 3 days per week), ‘often’ (the activity is done 4 to 6 days per week), and ‘always’ (the activity is done every day). The original version of KAP-PI instrument was written in Bahasa Indonesia using the standard vocabulary and structures of the Indonesian national language.

#### Content and face validity assessment

Two nurses and one physician from Indonesia who have experience in the field of pressure injuries and community care were asked to evaluate the content of the instrument. Experts received the draft instrument via email and were asked to rate 1) the clarity of wording per item and 2) the relevance of each item per domain (knowledge, attitude, and practice) to the population under consideration (family caregivers caring for community-dwelling older adults).They were asked to rate the items on a 4-point Likert scale as follows:

1. How do you assess the relevance of topics and items in the three domains for the population under consideration? Answer options included 1 = not relevant, 2 = somewhat relevant, 3 = quite relevant, 4 = highly relevant.
2. How do you assess the clarity of wording of this item? Answer categories were: 1 = drop item entirely, 2 = make major revisions to the item, 3 = make minor revision to the item, 4 = retain the item exactly as worded.

If experts felt that the wording was not clear or the item was not relevant, they were asked to suggest for improvements. In addition, the experts were allowed to add topics or points which, in their opinion, were still missing in the instrument. The item content validity index (I-CVI) was calculated to evaluate the individual items in the instrument. The I-CVI is computed as the number of experts giving a rating of either 3 (quite relevant) or 4 (highly relevant), divided by the number of experts [33]. It is recommended that the I-CVI should reach to 1.00 if the number of experts is less than five [34] Therefore, only if the I-CVI was 1.00, meaning that all experts gave a rating 3 (quite relevant) or 4 (highly relevant) on an item, the item was left in the instrument. This meant that the Instrument content validity index (S-CVI), calculated as the proportion of items from the original instrument rated as ‘quite relevant’ or ‘highly relevant’ by the experts was also 1 (reflecting excellent content validity) [33].

Next, face validity was assessed in the target population by including 12 family caregivers of community-dwelling older adults. This process aimed to confirm that family caregivers understood the questions as the researchers intended. They were first asked to fill out the instrument. Then, in a short interview, family caregivers were asked whether they recognized every word used on the questionnaire and understood the meaning of the question or not. Correction of sentences and word choices were made based on their feedback.

The content and face validity process resulted in a preliminary KAP-PI instrument.

### Phase II: Instrument construction

Phase II aimed to transform the preliminary KAP-PI instrument into a statistically acceptable construct [25]. At this stage, data were collected using a self-administration paper-based instrument and used to determine: 1) which items should be deleted; and 2) the optimal number of factors that fit a set of items [25].

#### Participants

For phase II, family caregivers were required to complete the preliminary version of the KAP-PI. For this, family caregivers of community-dwelling older adults in all regions in Bandung, Indonesia, were randomly selected from data provided by the municipalities. The data included information on the number, names, and addresses of families of all older adults in each area. Those data could be accessed after permission was obtained from the Health Department and the National Unity Agency, and Politics and Protection of the Regional People, which are the governmental institutions taking the responsibilities in health care and community protection in Indonesia.The number of family caregivers to be included in each community was determined by the ratio of older adult families to the total number of older adult families in all regions. A table of random numbers was used for selection. To be included in the study, family members (spouses, children, or other relatives of older adults) had to be living with or caring for people aged 60 years or older (considered older adults in Indonesia [35]). Each family member completed one instrument from each selected family, regardless of the number of older adults in their household. The number of participants needed for the statistical analysis purposes was determined by 10 participants per survey item or 200 – 300 participants [25].

#### Data collection

Selected family caregivers were visited in their home by enumerators, who were independently recruited as research assistants. If they met the inclusion criteria and agreed to participate, data collection immediately took place in the participant's home. Participants received an informed consent form and the Preliminary KAP-PI paper-based instrument consisting of demographic data, questions and instructions. The enumerators first explained the study's objectives, the procedure, the anonymized use of data and the right to stop participation at any moment. Participants completed the self-administration of preliminary KAP-PI instrument in front of the enumerators. The completed instruments were collected by the enumerators and given to the authors.

#### Data analysis

##### *Knowledge domain*

The answers to the questions, which consisted of multiple-choice questions, were recorded in dichotomous correct-incorrect variables. Correct answers were assigned the value 1, incorrect answers the value 0. Instrument construction of the knowledge domain was based on an evaluation described by Haladyna [31], which includes the evaluation of 1) the item difficulty; 2) the discriminating index (D value); and 3) the quality of response alternatives. The decision to delete some items was based on these ratings as explained below:

The item difficulty is the percentage of participants who answer the item correctly [31]. Items with a lower item difficulty are relatively easier to answer compared to items with a higher difficulty score. In this study, items answered correctly by less than 10% of the participants were considered too difficult and item answered correctly by more than 90% of the participants were considered too easy. Hence, items with a difficulty index lower than 0.10 or higher than 0.90 were removed.

The discriminating index (D value) describes an item’s ability to differentiate between participants who know and do not know the information being asked. A statistical method of Item-total correlation (point-biserial) was used to evaluate the discrimination index of each item. Further, Cronbach’s Alpha was checked. Items with item-total correlation being much lower than those of the other items and not contributing to internal consistency (i.e. alpha if item deleted > alpha with item in the scale) were deleted [31].

Finally, the quality of the response alternatives (that is, the distractors/wrong answers) was assessed by the proportion or percentage of participants who chose these distractors (range 0—1) [33]. The distractors with a value of 0 were defined as ‘not attractive’, and those with a value of 1 as ‘too attractive’. Response alternatives less than 0.10 or higher than 0.90 were modified or deleted.

A study of dimensionality from the valid items in the knowledge domain was performed using a Principal Factor Analysis (PFA) with oblique rotation method with Kaiser Normalization.

##### Attitude and practice domain

In the attitude and practice domains, the answers to the questions were scored according to each answer’s value, which ranged from 1 to 4. A Principal Factor Analysis (PFA) with oblique rotation method with Kaiser Normalization was run separately for the two domains. This was done to check the relationship between the items and to check how many factors were generated from the items [35, 36].

Before performing the factor analysis, all requirements for performing PFA were checked, including 1) value of Kaiser–Meyer–Olkin Measure from Sampling Adequacy (KMO MSA) should be 0.50; 2) Bartletts Test of sphericity (Sig.) should be 0.05 [37]. The number of factors in the attitude and practice domains were extracted using Eigenvalues > 1 and the total variance explained by the factors [37]. A factor loading cutoff value of 0.4 was used to indicate the acceptable construct validity of each item: items with a value of 0.4 and higher were retained in the instrument [36].

All analyses in phase II were done using IBM SPSS Statistics 26 (IBM Corp, Armonk, NY).

### Phase III: Psychometric testing of the instrument

#### Participants and procedure

Phase III tested the final KAP-PI instrument to different family caregivers in the same population with phase II. The inclusion criteria and handling of the data collection procedure for phase III were the same as phase II. The number of participants needed was determined by 10 participants per survey item or 200 – 300 participants [25].

#### Data analysis

In the knowledge domain, data analysis aimed to confirm the dimensionality of items. All the valid items were expected to form a unidimensional construct. In the attitude and practice domains, data analysis aimed to confirm number of factors constructed from phase II. A confirmatory factor analysis (CFA) was performed using the statistical computing R package lavaan from the Comprehensive R Archive Network (CRAN) [38, 39]. The R package lavaan generates “fit indices” including Tucker-Lewis Index/ TLI (the higher the value, the better the model), Root Mean Square Error of Approximation/ RMSEA ( is expected to be small to indicate reasonable model fit), and Comparative Fit Index/ CFI (value above 0.90 is considered good) [40]. When the indices are fits, the correct model has been specified [40].

Lastly, the instrument's reliability (internal consistency) in each domain and per item subset were analyzed using Cronbach’s alpha inter-item correlation. A general guideline for the use of Cronbach's alpha to assess a newly developed instrument is that values should be ≥ 0.70 [41].

#### Ethics approval and informed consent

All methods were performed in accordance with the relevant guidelines and regulations of the Declaration of Helsinki, a statement of ethical principles which directs research involving human subjects. This study received ethical approval from The Research Ethics Committee Universitas Padjadjaran Bandung (No. 138 / UN6.KEP/EC/2020). Furthermore, two governmental institutions that have responsibilities in health care and community protection approved the research project before undertaken (the Health Departement #070/13472-Dinkes and the National Unity Agency, Politics and Protection of the Regional People #070/3177/Bakesbangpol). Participants received information about the study and signed consent if they agreed to participate. Participants were not obligated to participate and could refuse participation before and during the data collection. Informed consent was obtained from all participants.

## Results

### Characteristics of participants

Table 1 shows the participant characteristics. A total of 372 family caregivers participated in the study (12 participants in Phase I to investigate face validity, 120 participants in Phase II for instrument development, and 240 participants in Phase III for psychometric testing). Of all participating family caregivers, 89.0% (*n* = 331) were female, and 69.1% (*n* = 257) were > 30 years old. Most participants (*n* = 307; 82.5%) had a low educational background (below upper secondary school education), and 38.9% (*n* = 144) were unemployed. More than half (*n* = 266; 71.5%) of the caregivers were children of older adults.

**Table 1:** Characteristics of participants

**Characteristics of Participants**	**Participants (%)**			
	**Face validity (*n* = 12)**	**Phase II (*n* = 120)**	**Phase III (*n* = 240)**	**Total (*n* = 372)**
Gender				
Male	3 (20.0)	15 (12.5)	23 (9.6)	41 (11.0)
Female	9 (80.0)	105 (87.5)	217 (90.4)	331(89.0)
Age category				
< 20 years	0 (0.0)	7 (5.8)	14 (5.8)	21 (5.6)
20 – 30 years	3 (20.0)	28 (23.3)	63 (26.3)	94 (25.3)
31 – 40 years	6 (60.0)	33 (27.5)	54 (22.5)	93 (25.0)
> 40 years	3 (20.0)	52 (43.3)	109 (45.4)	164 (44.1)
Education				
Primary education	3 (30.0)	82 (68.3)	147 (61.3)	232 (62.4)
Lower secondary education	2 (10.0)	15 (12.5)	58 (24.2)	75 (20.2)
Upper secondary education	7 (60.0)	16 (13.3)	27 (11.3)	50 (13.4)
Diploma	0 (0.0)	7 (5.8)	8 (3.3)	15 (4.0)
Occupation				
Unemployed	4 (40.0)	48 (40.0)	92 (38.3)	144 (38.7)
Student	0 (0.0)	2 (1.7)	3 (1.3)	5 (1.3)
Employee	2 (20.0)	38 (31.7)	84 (35.0)	124 (33.3)
Self-employed	6(40.0)	32 (26.7)	61 (25.4)	99 (26.6)
Relationship with older adult				
Spouse	0 (0.0)	17 (14.2)	28 (11.7)	45 (12.1)
Children	6 (50.0)	63 (52.5)	170 (70.8)	239 (64.2)
Other relatives	6 (50.0)	40 (33.3)	42 (17.5)	88 (23.7)

### Knowledge domain

Table 2 shows the topics and items generated in the knowledge domain and their statistical analysis results at each stage. In the process of item development, four topics and 18 items were generated (items no. 1—18). In the process of content validation, of these 18 items, five items (item no. 2, 5, 9, 12, and 18) were deleted due to a Content Validity Index (CVI) of < 1 each, while one item was added to the supporting interface (item no. 19). Thus, 14 items remained content valid and therefore, were tested for construct validity in phase II.

**Table 2:** Knowledge domain

No	Domain and item generation	Phase I		Phase II				Phase III		
		**I-CVI**	**Conclusion**	**Item difficulty**	**Discriminating index**	**Quality of response alternatives**	**Conclusion**	**Factor loading**	**“fit indices” of CFA**	**Cronbach’s Alpha**
**Topic: Definition and characteristic of older adult**									Multifocal model with three factors CFI = 0.87 TLI = 0.82 RMSEA = 0.04	Overall: 0.83 F1: 0.77 F2: 0.78 F 3: 0.74
**1**	Older adults in Indonesia is defined as: a. People aged ≥ 50 years b. People aged ≥ 60 years* c. People aged ≥ 70 years	**1**	Retained	0.40	**0.09**^**c**^ Cronbach’s Alpha if item deleted = 0.85	Options: a = 0.25 c = 0.35	**Deleted**	Item deleted/ not tested		
**2**	What is characteristic of older adults: a. Have limited regenerative abilities* b. Have limited activity daily living c. Have limited interaction with people	** < 1**	Deleted	Item deleted/ not tested	Item deleted/ not tested	Item deleted/ not tested	Item deleted/ not tested	Item deleted/ not tested		
**3**	The normal changes that occur in the older adult’s skin are: a. The skin becomes wrinkled and moist b. The skin becomes wrinkled and gets wet easily c. The skin becomes wrinkled and dries easily*	**1**	Retained	0.72	0.63 Cronbach’s Alpha if item deleted = 0.81	Options: a = 0.19 b = 0.17	Retained	**0.58 (factor 3)**		
**Topic: Definition of PI and symptoms associated with PIs**										
**4**	A Pressure injury is: a. An injury that occurs due to the use of diapers b. An injury on the skin which usually occurs over a bony prominence as a result of pressure* c. An injury that occurs due to pressed by tight clothes	**1**	Retained	0.30	0.50 Cronbach’s Alpha if item deleted = 0.82	Options: a = 0.50 c = 0.20	Retained	**0.47 (factor 3)**		
**5**	A pressure injury can also be defined as: a. An injury on the skin over a bony prominence as a result of shear b. An injury on the skin because of heat c. An injury on the skin because of diabetic	** < 1**	Deleted	Item deleted/ not tested	Item deleted/ not tested	Item deleted/ not tested	Item deleted/ not tested	Item deleted/ not tested		
**6**	Symptom(s) of pressure injuries are: a. The skin looks reddish b. There is visible skin damage/wounds c. Options A and B are correct*	**1**	Retained	0.72	0.68 Cronbach’s Alpha if item deleted = 0.80	Options: a = 0.18 b = 0.10	Retained	**0.42 (factor 3)**		
**Topic: Cause and consequences of PIs**										
**7**	The cause of a pressure injury is: a. Continuous pressure and shear against the skin* b. Squeezed objects falling on the body c. The pressure of clothes attached to the body	**1**	Retained	0.53	0.54 Cronbach’s Alpha if item deleted = 0.81	Options: b = 0.13 c = 0.34		**0.67 (factor 2)**		
**8**	Pressure injuries in older adults can cause: a. Pain and infection* b. Nausea and vomiting c. Urinary incontinence	**1**	Retained	0.67	0.62 Cronbach’s Alpha if item deleted = 0.81	Options: b = 0.14 c = 0.19	Retained	**0.48 (factor 2)**		
**9**	Pressure injuries in older adults can make them: a. Difficult to mobile* b. Difficult to urinate c. Difficult to concentrate	** < 1**	Deleted	Item deleted/ not tested	Item deleted/ not tested	Item deleted/ not tested	Item deleted/ not tested	Item deleted/ not tested		
**10**	What will happen if redness in the skin of older adults is left untreated? a. It will develop into deep pressure ulcers* b. It becomes blackish and then heals c. It will heal itself when the skin is dry	**1**	Retained	**0.08**^**b**^	**0.36**^**c**^ Cronbach’s Alpha if item deleted = 0.83	Options: b = 0.32 c = 0.60	**Deleted**	Item deleted/ not tested		
**Topic: Preventive strategies that family caregivers can perform to prevent PIs**										
**11**	What to do to prevent pressure injuries in older adults? a. Wear loose clothes b. Use footwear when leaving the house c. Prevent prolonged pressure on the skin*	**1**	Retained	0.38	0.49 Cronbach’s Alpha if item deleted = 0.82	Options: a = 0.42 b = 0.20	Retained	**0.44 (factor 1)**		
**12**	What to do to prevent redness on older adult’s skin? a. Prevent shear on the skin b. Prevent applying lotion on the skin c. Prevent using hard mattress	** < 1**	Deleted	Item deleted/ not tested	Item deleted/ not tested	Item deleted/ not tested	Item deleted/ not tested	Item deleted/ not tested		
**13**	Pressure ulcers in older adults can also be prevented by: a. Adequate feeding and drinking* b. Sunbathing c. Prevent stress on older adults	**1**	Retained	0.66	0.62 Cronbach’s Alpha if item deleted = 0.81	Options: b = 0.13 c = 0.21	Retained	**0.92 (factor 1)**		
**14**	For immobile/ bedridden older adults, what should be done to prevent pressure injuries? a. Mobilization to the left and right sleeping position* b. Positioning the older adults always sleeps on their back without any wedge c. Let older adults sleep without being disturbed	**1**	Retained	0.49	0.58 Cronbach’s Alpha if item deleted = 0.81	Options: b = 0.31 c = 0.20	Retained	**0.95 (factor 1)**		
**15**	A thing that should be done on older persons' dry skin to avoid pressure injuries is: a. Apply powder to keep the skin dry b. Moisturizes dry skin* c. Cover the dry skin with a bandage	**1**	Retained	0.53	0.60 Cronbach’s Alpha if item deleted = 0.81	Options: a = 0.23 c = 0.24	Retained	**0.44 (factor 1)**		
**16**	A thing that should be done when an older persons' skin turns red is: a. Let it dry itself b. Release pressure and shear* c. Give betadine or iodine	**1**	Retained	0.75	0.70 Cronbach’s Alpha if item deleted = 0.81	Options: a = 0.15 c = 0.10	Retained	**0.45 (factor 1)**		
**17**	A thing that should be done if the skin of an older adult shows deep pressure injury is: a. Take the older adults to health care services* b. Treat using honey c. Let it open	**1**	Retained	0.71	0.59 Cronbach’s Alpha if item deleted = 0.81	Options: b = 0.17 c = 0.12	Retained	**0.95 (factor 1)**		
**18**	What to do to prevent deep pressure injuries in older adults: a. Consult the injuries to health care provider* b. Apply a traditional medicine like honey or coffee c. I do not know the answer	** < 1**	Deleted	Item deleted/ not tested	Item deleted/ not tested	Item deleted/ not tested	Item deleted/ not tested	Item deleted/ not tested		
**19**	Using a special mattress for older adults can prevent pressure injuries. This statement is: a. True* b. False c. I do not know	**1**	Added	0.49	0.58 Cronbach’s Alpha if item deleted = 0.81	Options: b = 0.36 c = 0.15	Retained	**0.92 (factor 1)**		

Note:

^*^correct answers

^a^Value of person correlation (r-value) smaller than r-table

^b^Value of item difficulty smaller than 0.10 or larger than 0.90

^c^Value of discriminant index (D value) smaller than 0.40

**Fig. 2:**
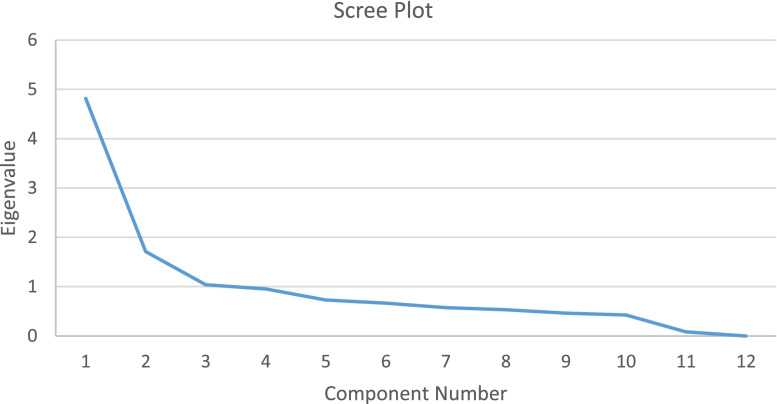
Scree plot resulted from principal factor analysis (PFA) in knowledge domain

In the data analysis of phase II, two items (items no.1 and no.10) were identified who had item-total correlation much lower than those of the other items. These two items did not contributed to the Cronbach’s alpha; means that if these two items were deleted, the Cronbach’s alpha was higher. Item no.10 also had a difficulty index < 0.10. As a result, item no.1 and 10 were removed from the instrument, leaving 12 items with a good difficulty index (mean = 0.57), good discriminating index (mean = 0.59) and good distractors of the multiple-choice alternatives. The dimensionality of all 12 items were tested using Principal Factor Analysis (PFA), generating three factors for the Eigenvalue greater than 1 (as shown in Fig. 2). Factor 1 represented topic about PI prevention (item no.6,7,8,9,10), factor 2 was about cause and consequences of PIs (item no.7 and 8), and factor 3 related to characteristic of older adults and PI (item no.1,2,3). These three factors together explained 64.4% of total variance. Factor loading of these 12 items ranged from 0.40–0.92.

A Confirmatory Factor Analysis with three factor was run for these 12 items in phase III among 240 family caregivers. The results showed that a model of three factor was accepted (CFI = 0.87; TLI = ; 0.82 and RMSEA = 0.04). The internal consistency of Cronbach's alpha was 0.83. All these results show that the KAP-PI instrument in the knowledge domain with 12 items can be used as an instrument to measure family caregivers' knowledge prevention among community-dwelling older adults.

### Attitude domain

Table 3 shows the items generated in the item development and psychometric evaluation of the items in the attitude domain. At the beginning, ten items (items no.1–10) were generated. Based on the content validity, two (items no.2 and 6) of these items were deleted due to the CVI < 1 of each, while one item was added to the supporting interface (item no.11), leaving nine items to be included in phase II.

**Table 3:** Attitude domain

No	Domain and item generation	Phase I	Phase II		Phase III		
			**PFA (*n* = 120) Factor Loading**	**Reliability (Cronbach’s Alpha)**	**CFA (*n* = 240) Factor Loading**	**Reliability (Cronbach’s Alpha)**	**“fit indices” of CFA**
1	I am responsible for the health of the older relative in my house	Retained (I-CVI = 1)	0.69	0.92	0.66	0.93	Tucker-Lewis Index/ TLI = 0.83 Comparative Fit Index/ CFI = 0.87 RMSEA = 0.02
2	I am responsible for pressure injury problems in the older relative in my house	Deleted (I-CVI < 1)	Item deleted/ not tested		Item deleted/ not tested		
3	The personal hygiene of the older relative in my house must be cared for carefully	Retained (I-CVI = 1)	0.71		0.68		
4	I have to pay attention to the skin moisture and hygiene of the older relative in my house	Retained (I-CVI = 1)	0.79		0.75		
5	It is important to pay attention to the food and drink of the older relative in my house	Retained (I-CVI = 1)	0.88		0.83		
6	I am responsible for the nutritional problem in the older relative in my house	Deleted (I-CVI < 1)	Item deleted/ not tested		Item deleted/ not tested		
7	Pressure injuries on the older relative in my house should be prevented	Retained (I-CVI = 1)	0.87		0.86		
8	Helping the older relative in my house in their activities and movements is my responsibility	Retained (I-CVI = 1)	0.79		0.81		
9	Immobile older relative in my house need to be helped in movement and positioning	Retained (I-CVI = 1)	0.77		0.77		
10	The older relative in my house who experience pressure injuries need to be checked to health care service	Retained (I-CVI = 1)	0.80		0.80		
11	The older relative in my house who are at risk of getting pressure injuries need a special mattress to prevent pressure injuries	Added (I-CVI = 1)	0.78		0.75		

**Fig. 3:**
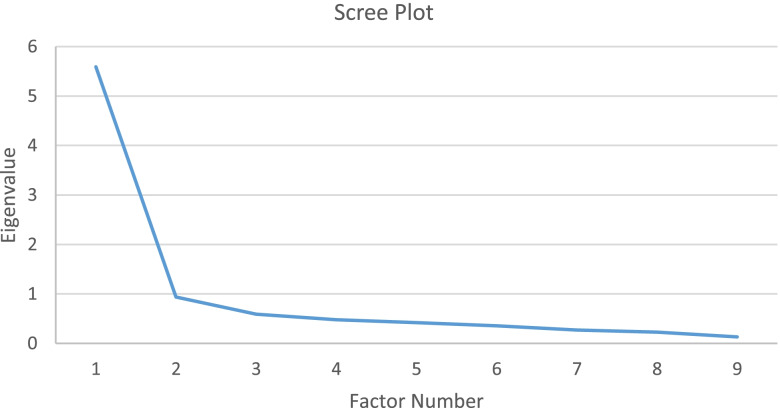
Scree plot resulted from principal factor analysis (PFA) in attitude domain

A Principal Factor Analysis (PFA) with oblique rotation was run for phase II. All requirements for performing PFA were met (KMO MSA = 0.89; Bartletts Test of sphericity (Sig.) = 0.00). All nine items tested had factor loading > 0.40 of each (0.64 – 0.87), which means that no items had to be deleted. As shown in Fig. 3, these nine items constructed one factor for the Eigenvalue greater than 1 and explained 62.13% of the total variability, which is higher than the required 60% [42]).

In phase III, all nine items were tested among 240 family caregivers. A Confirmatory Factor Analysis was run resulting in “fit indices” including Tucker-Lewis Index/ TLI of 0.83; Comparative Fit Index/ CFI of 0.87; and RMSEA of 0.02. These three “fit indices” indicated that the unidimensional model which resulted from the PCA in phase II, was confirmed as the model fit. The internal consistency of the final versions of the KAP-PI instrument in the attitude domain had a Cronbach's alpha of 0.93, indicating high reliability. In conclusion, all validity and reliability test results indicate that the nine items can be trusted as a means of measuring family caregivers' attitudes toward pressure injury prevention in community-dwelling older adults.

### Practice domain

**Fig. 4:**
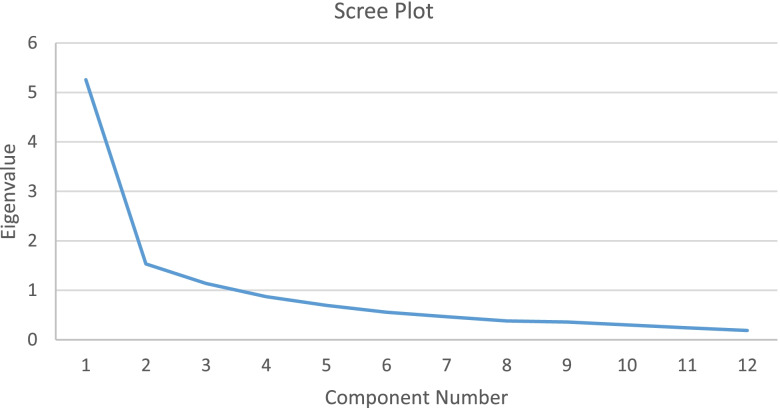
Scree plot resulted from principal factor analysis (PFA) in practice domain

Table 4 shows the items generated in the practice domain and its psychometric evaluation. Four topics and 13 items (items no.1–13) were developed in phase I. Of the 13 items, one item (item no.13) was deleted were deleted due to the CVI < 1 of each, while one item was added to the supporting interface (item no.14), leaving 13 items to be tested for phase II. A PFA with oblique rotation was run in phase II. All requirements for performing PFA were met (KMO MSA = 0.85; Bartletts Test of sphericity (Sig.) = 0.00). When looking to the factor loading of each item, item no.12 had a factor loading < 0.40 (0.14), indicating the item should be deleted from the instrument. After deleting item no.12, the PFA with oblique rotation was re-run and this second PFA resulted in a factor loading > 0.40 of each item (0.41 – 0.82). As shown in Fig. 4, all 12 items constructed three factors for the Eigenvalue greater than 1, i.e. factor 1 (item no. 6, 7, 8,and 9), factor 2 (item no.1, 2, 10, 11, and 12), and factor 3 (item no. 3, 4, and 5). These three factors explained 66.11% of the total variability (required not less than 60% [42]).

**Table 4:** Practice domain

No	Domain and item generation	Phase I	Phase II				Phase III				
			**PFA (*n* = 120) Factor Loading**			**Reliability (Cronbach’s Alpha)**	**CFA (*n* = 240) Factor Loading**			**Reliability (Cronbach’s Alpha)**	**Fit indices of CFA**
			**Factor 1**	**Factor 2**	**Factor 3**		**Factor 1**	**Factor 2**	**Factor 3**		
	**Topic: Activities performed to support older adults to meet their nutritional and fluid needs**					**0.87**				**Overall:** **0.89** **Factor 1:** 0.80 Factor 2: 0.73 Factor 3: 0.87	Tucker-Lewis Index/ TLI = 0.83 Comparative Fit Index/ CFI = 0.87 RMSEA = 0.06
1	Provide healthy food for the older relative in my house	Retained (I-CVI = 1)		0.74				0.51			
2	Provide mineral water for the older relative in my house at least 8 glasses in a day	Retained (I-CVI = 1)		0.53				0.55			
	**Topic: Activities performed to support older adults in mobilization and repositioning**										
3	Helping the older relative in my house to do activities if they cannot do it him/herself	Retained (I-CVI = 1)			0.86				0.87		
4	Helping for the older relative in my house to move if they cannot do it him/herself	Retained (I-CVI = 1)			0.78				0.82		
5	Helping the bedridden older relative in my house to change their position (positioning) regularly if they cannot do it him/herself	Retained (I-CVI = 1)			0.61				0.87		
	**Topic: Activities performed to support older adults in skin hygiene and moisture care**										
6	Prevent the older relative in my house from using damp and wet clothes, including changing diapers regularly (if they use diapers)	Retained (I-CVI = 1)	0.75				0.64				
7	Prevent long pressure on the body of the older relative in my house	Retained (I-CVI = 1)	0.85				0.87				
8	Moisturizing the skin of the older relative in my house by giving lotions/oils	Retained (I-CVI = 1)	0.76				0.85				
9	Check the entire skin of the older relative in my house for redness	Retained (I-CVI = 1)	0.71				0.67				
	**Topic: Activities performed to support older adults to maintain their health and** ensure access to health care services										
10	Maintain the environmental hygiene of for the older relative in my house	Retained (I-CVI = 1)		0.68				0.67			
11	Took the older relative in my house to health services if they suffer from wounds	Retained (I-CVI = 1)		0.61				0.74			
12	Took the older adult to health services if they had a health problem	Retained (I-CVI = 1)	MSA = 0.45 (< 0.50) Item was not included in PCA				Item deleted/ not tested				
13	Contact health care provider to get their suggestion according to condition of the older relative	Deleted (I-CVI < 1)	Item deleted/ not tested				Item deleted/ not tested				
	**Topic: Support older adult to have a special mattress**										
14	Provide a special mattress for a bedridden elderly relative in my house	Added (I-CVI = 1)			0.64			0.49			

Only the highest factor loading is shown

In phase III, all 12 items were tested among 240 family caregivers. A Confirmatory Factor Analysis was run to check whether the three factors resulted by Principal Factor Analysis (PFA) was a good model or not. The CFA resulted in “fit indices”, i.e. Tucker-Lewis Index/ TLI of 0.83; Comparative Fit Index/ CFI of 0.87; and RMSEA of 0.06. These three “fit indices” indicated that the three factor model resulting from the PCA in phase II, was confirmed as the model fit for practice domain. The correlations between factors were > 0.60. The internal consistency a Cronbach's alpha of factor 1, 2 and 3 were 0.80, 0.73, and 0.87 respectively. The overall internal consistency of the final version of the KAP-PI instrument in the practice domain had a Cronbach's alpha of 0.89, indicating high reliability. In conclusion, all validity and reliability test results indicate that the 12 items can be trusted as a means of measuring family caregivers' practice toward pressure injury prevention in community-dwelling older adults.

## Discussion

In this study, following a guideline described by Boateng et al. [25], an instrument to measure knowledge, attitude, and practice of family caregivers to prevent PIs among community-dwelling older adults (KAP-PI) in Indonesia was developed and psychometrically tested. The results demonstrate that the final version of the KAP-PI was valid and had Cronbach’s Alpha values of 0.83, 0.93 and 0.89 in the respective knowledge, attitude and practice domains. This also indicates high reliability.

Background characteristics of the sample show heterogeneity with regard to gender, age group, educational level, employment and relationship to the community-dwelling older adult. This sample is a good representation of the targeted population of family caregivers in Indonesia who mostly care for their parents or relative at home (extended family). Irrespective of the fact that the group was highly heterogeneous, the KAP-PI instrument showed good performance in the statistic analysis [31].

In the final version of the KAP-PI instrument, the knowledge domain consists of 12 items. To assess the validity of the items, item difficulty, discriminating index, and quality of response alternatives were measured. These measurements are commonly used in studies focused on developing knowledge questionnaires, such as described by Beeckman et al. (2010) and Manderlier et al. (2017), who developed an instrument to measure nurse’s knowledge about PI prevention that had been used in many studies [43–46]. Even though our target group, family caregivers, is different from those described in these studies (nurses), it is important that family caregivers know definition, symptoms, and consequences of PIs prior to knowledge about activities needed to prevent PI [19, 28]. These topics were not covered in a prior study by Bellon and Pancarbo [24], who also developed and tested a questionnaire to assess family caregiver's knowledge about PI prevention. Additional topics about definition, symptoms, and consequences of PIs were included in the KAP-PI instrument. However, study by Bellon and Pancarbo and our study covered almost the same points of knowledge about activities of PI prevention. Furthermore, construct evaluation of the knowledge domain of the KAP-PI instrument generated three factors. Hence, when using the KAP-PI instrument in a real survey, the results should be analyzed for each factor.

When looking at the attitude domain, the nine items are highly correlated, constructing the unidimensionality of the attitude domain of the KAP-PI instrument. Attitude is an affective aspect of a person that causes him or her to take a certain action [47]. Measuring attitude is essential if a specific behavior or practice is an outcome of the intervention; for example, when looking at health education as an intervention you hope to see different behavior afterwards [23, 48]. In this study, the attitude domain reflects the beliefs and values of family caregivers towards PI prevention. The most important aspect of attitude is the willingness of family caregivers to support older adults in preventing PIs. The willingness to help others is an essential factor for family caregivers and any informal caregivers before being involved in an education or empowerment program [49–52]. In family nursing practice, affection is a binding domain that should be considered when planning and performing a family nursing intervention [27]. Several studies have developed instruments in affective or emotional domains to assess family functioning among patients with different conditions. For example, the Iceland-Expressive Family Functioning Questionnaire (ICE-EFFQ) measuring expressive family functioning when experiencing acute or chronic illnesses [53]. The ICE-EFFQ was psychometrically tested using the same data analysis techniques (EFA followed by CFA) as used in our study. The KAP-PI instrument measures the affective aspect of family caregivers in PI prevention. This current study added inventory family affective assessment tools to prior published instruments such as Family functioning, Health, and Social Support (FAFHES) used for a family of an adult cardiac patient [54] and Family Assessment Device (FAD) measuring family functioning in general [55, 56].

In the practice domain, three topics emerged and were validated: basic support, reposition and mobilization support, and skin hygiene and moisture support. The Likert scale was used to assess family caregiver activities about PI prevention for their older relatives. Nurses working with families, which in Indonesia usually performed by community nurses, can use the KAP-PI instrument to get insight to what extent family caregivers actually perform the essential activities to prevent PIs in their relatives. Nurses should consider the nature of family caregivers’ support for their older relatives [57]. By assessing family caregiver’s knowledge, attitude, and practice using the KAP-PI instrument, nurses can arrange a training program to increase families' competencies in their authority as informal caregivers to prevent PI in their older relatives. Considering that the knowledge and practice domain consists of three sub-variables when interpreting the results, nurses can critically analyze which sub-variable the family caregiver has the lower score on and then prioritize their intervention based on the results. Although test–retest reliability was not established, the current study obtained high values for Cronbach's alpha in both studies (phases 2 and 3), meaning that the KAP-PI instrument can be used directly either in practice or research purposes.

### Limitations

One limitation of this study could be that the content validity was based on only three experts. To account for the limited number of included experts, only the items rated quite relevant or highly relevant by all experts were retained in the instrument. Also, a thorough statistical analysis was done in phase 2 and 3 to ensure validity and reliability of the instrument. Another limitation is that test–retest reliability was not determined. However, validity and reliability were derived from two independent samples from two different data collection procedures, including relatively large sample size (120 participants in phase II and 240 participants in phase III), obtaining high values for Cronbach's alpha in both studies. Therefore, we believe the instrument was thoroughly developed and is good to use among our intended population.

## Conclusion

An instrument to assess knowledge, attitude and practice of Family Caregiver on Preventing Pressure Injuries (KAP-PI) among community-dwelling older adults in Indonesia was developed and validated. A 12-item knowledge domain, a 9-item attitude domain, and a 12-item practice domain were designed based on a guided construction process. The validity and reliability of the instrument were statistically acceptable. The instrument can be used directly in family nursing practice, education, and research to assess the function of family caregivers in preventing pressure injuries among community-dwelling older people in Indonesia.

## Supplementary Information

**Additional file1.** 

## Data Availability

All data generated or analysed during this study are included in this published article [and its supplementary information file].

## Abbreviations

CFA: Confirmatory Factor Analysis
KAP_PI: Knowledge, Attitude and Practice of family caregivers to prevent PIs among community-dwelling older adults in Indonesia
I-CVI: Item content validity index
NPUAP: National Pressure Ulcer Advisory Panel
PFA: Principal Factor Analysis
PI: Pressure Injury
PPPIA: Pan Pacific Pressure Injury Alliance
S-CVI: Instrument content validity index
UPUAP: European Pressure Ulcer Advisory Panel
