# Supplementary material for: Development and psychometric evaluation of an instrument to assess Knowledge, Attitude and Practice of Family Caregivers at Preventing Pressure Injuries (KAP-PI) in Indonesian community-dwelling older adults
Source: BMC Nurs. 2022 Aug 11;21:222. doi: 10.1186/s12912-022-00957-4 (PMC9367027; doi:10.1186/s12912-022-00957-4)
Supplement: Supplementary file 1 — Additional file1. [file 12912_2022_957_MOESM1_ESM.docx]

Development and Psychometric Evaluation of an Instrument to Assess Knowledge, Attitude and Practice of Family Caregivers at Preventing Pressure Injuries (KAP-PI) in Indonesian Community-Dwelling Older Adults

**(The instrument)**

1. **Characteristics of Family Caregivers**

| Age : |
| --- |
| Sex : |
| Age of your older relative: |
| Educational Background |
| 1. Primary education |
| 1. Lower secondary education |
| 1. Upper secondary education |
| 1. Bachelor or higher |
| Occupation |
| 1. Unemployed |
| 1. Student |
| 1. Employee |
| 1. Self-employed |
| Relationship with older adults |
| 1. Children |
| 1. Spouse |
| 1. Other relatives, mentioned: |
| Living in the same house with older adults |
| Yes No |
| Sharing responsibility with other in caring for older adults |
| Yes No |
| Duration of caring for older adults |
| 1. ≤ 1 year |
| 1. > 1 - ≤ 3 years |
| 1. > 3 - ≤ 5 years |
| 1. > 5 years |
| Duration of supporting older adult’s ADL in a day |
| 1. Undefined |
| 1. ≤ 3 hours |
| 1. > 3 - ≤ 8 hours |
| 1. > 8 hours |
| Living with older adult (s) who experienced PI(s) |
| Yes No I do not know |
| Had experience in PI care |
| Yes No |

1. **Knowledge domain**

Instructions :

1. Choose one of the correct answers from the answers below.
2. If you find the word “older adults or older relatives”, it refers to people aged 60 years or older.

| **Topic: Definition of PI and older adult’s characteristics** | |
| --- | --- |
| **1** | The normal changes that occur in the older adult’s skin are:   1. The skin becomes wrinkled and moist 2. The skin becomes wrinkled and gets wet easily 3. The skin becomes wrinkled and dries easily* |
| **2** | A Pressure injury is:   1. An injury that occurs due to the use of diapers 2. An injury on the skin which usually occurs over a bony prominence as a result of pressure* 3. An injury that occurs due to pressed by tight clothes |
| **Topic: Symptoms, cause, and consequences of PI** | |
| **3** | Symptom(s) of pressure injuries are:   1. The skin looks reddish 2. There is visible skin damage/wounds 3. Options A and B are correct* |
| **4** | The cause of a pressure injury is:   1. Continuous pressure and shear against the skin* 2. Squeezed objects falling on the body 3. The pressure of clothes attached to the body |
| **5** | Pressure injuries in older adults can cause:   1. Pain and infection* 2. Nausea and vomiting 3. Urinary incontinence |
| **Topic: Preventive strategies that family caregivers can perform to prevent PIs** | |
| **6** | What to do to prevent pressure injuries in older adults?   1. Wear loose clothes 2. Use footwear when leaving the house. 3. Prevent prolonged pressure on the skin* |
| **7** | Pressure injuries in older adults can also be prevented by:   1. Adequate feeding and drinking* 2. Sunbathing 3. Prevent stress on older adults |
| **8** | For immobile/ bedridden older adults, what should be done to prevent pressure injuries?   1. Regularly mobilize the older adults to the left and right sleeping position* 2. Positioning the older adults always sleeps on their back without any wedge 3. Let older adults sleep without being disturbed. |
| **9** | A thing that should be done on older persons’ dry skin to avoid pressure injuries is:   1. Apply powder to keep the skin dry 2. Moisturizes dry skin* 3. Cover the dry skin with a bandage |
| **10** | A thing that should be done when an older persons’ skin turns red is:   1. Let it dry itself 2. Release pressure and shear* 3. Give betadine or iodine. |
| **11** | A thing that should be done if the skin of an older adult shows deep pressure injury is:   1. Take the older adults to health care services* 2. Treat using honey 3. Let it open |
| **12** | Using a special mattress for older adults can prevent pressure injuries. This statement is:   1. True* 2. False 3. I do not know |

1. **Attitude domain**

Would you please choose the correct answer to each of the following statements according to your opinion

| Statements | Strongly disagree | Disagree | Agree | Strongly agree |
| --- | --- | --- | --- | --- |
| I am responsible for the health of the older relative in my house |  |  |  |  |
| The personal hygiene of the older relative in my house must be cared for carefully |  |  |  |  |
| I have to pay attention to the skin moisture and hygiene of the older relative in my house. |  |  |  |  |
| It is important to pay attention to the food and drinks of the older relative in my house |  |  |  |  |
| Pressure injuries on the older relative in my house should be prevented |  |  |  |  |
| Helping the older relative in my house in their activities and movements is my responsibility |  |  |  |  |
| Immobile older relative in my house need to be helped in movement and positioning |  |  |  |  |
| The older relative in my house who experiences pressure injuries need to be checked by formal health care service |  |  |  |  |
| The older relative in my house who is at risk of getting pressure injuries needs a special mattress to prevent pressure injuries |  |  |  |  |

1. **Practice domain**

Instruction:

Please choose,

- Always if it was done every day in the last month
- Often if it was done 4 to 6 days a week in the last month
- Sometimes if it was done 1 to 3 days a week in the last month
- Never if it has never been done in the last month

| **Domain and item generation** | **Never** | **Sometimes** | **Often** | **Always** |
| --- | --- | --- | --- | --- |
| **Topic: Activities performed to support older adults to meet nutritional and fluid needs, maintain environmental hygiene, and access health care services** | | | | |
| Provide healthy food for the older relative in my house |  |  |  |  |
| Provide mineral water for the older relative in my house at least 8 glasses in a day |  |  |  |  |
| Maintain the environmental hygiene for the older relative in my house |  |  |  |  |
| Took the older relative in my house to health services if they suffer from wounds |  |  |  |  |
| **Topic: Activities performed to support older adults in**  **Mobilization, repositioning and facilitating a special mattress** | | | | |
| Helping the older relative in my house to do activities if they cannot do it him/herself |  |  |  |  |
| Helping for the older relative in my house to move if they cannot do it him/herself |  |  |  |  |
| Helping the bedridden older relative in my house to change their position (positioning) regularly if they cannot do it him/herself |  |  |  |  |
| Provide a special mattress for a bedridden elderly relative in my house |  |  |  |  |
| **Topic: Activities performed to support older adults in skin**  **hygiene and moisture care** | | | | |
| Prevent the older relative in my house from using damp and wet clothes, including changing diapers regularly (if they use diapers) |  |  |  |  |
| Prevent long pressure on the body of the older relative in my house |  |  |  |  |
| Moisturizing the skin of the older relative in my house by giving lotions/oils |  |  |  |  |
| Check the entire skin of the older relative in my house for redness |  |  |  |  |
